# Effect of NaCl stress on exoproteome profiles of *Bacillus amyloliquefaciens* EB2003A and *Lactobacillus helveticus* EL2006H

**DOI:** 10.3389/fmicb.2023.1206152

**Published:** 2023-08-28

**Authors:** Judith Naamala, Sowmyalakshmi Subramanian, Levini A. Msimbira, Donald L. Smith

**Affiliations:** Department of Plant Science, McGill University, Montreal, QC, Canada

**Keywords:** PGPM, *Lactobacillus helveticus*, *Bacillus amyloliquefaciens*, salt stress, exoproteome profile

## Abstract

Salt stress can affect survival, multiplication and ability of plant growth promoting microorganisms to enhance plant growth. Changes in a microbe’s proteome profile is one of the mechanisms employed by PGPM to enhance tolerance of salt stress. This study was focused on understanding changes in the exoproteome profile of *Bacillus amyloliquefaciens* EB2003A and *Lactobacillus helveticus* EL2006H when exposed to salt stress. The strains were cultured in 100 mL M13 (*B. amyloliquefaciens*) and 100 mL De man, Rogosa and Sharpe (MRS) (*L. helveticus*) media, supplemented with 200 and 0 mM NaCl (control), at pH 7.0. The strains were then incubated for 48 h (late exponential growth phase), at 120 rpm and 30 (*B. amyloliquefaciens*) and 37 (*L. helveticus*) °C. The microbial cultures were then centrifuged and filtered sterilized, to obtain cell free supernatants whose proteome profiles were studied using LC–MS/MS analysis and quantified using scaffold. Results of the study revealed that treatment with 200 mM NaCl negatively affected the quantity of identified proteins in comparison to the control, for both strains. There was upregulation and downregulation of some proteins, even up to 100%, which resulted in identification of proteins significantly unique between the control or 200 mM NaCl (*p* ≤ 0.05), for both microbial species. Proteins unique to 200 mM NaCl were mostly those involved in cell wall metabolism, substrate transport, oxidative stress tolerance, gene expression and DNA replication and repair. Some of the identified unique proteins have also been reported to enhance plant growth. In conclusion, based on the results of the work described here, PGPM alter their exoproteome profile when exposed to salt stress, potentially upregulating proteins that enhance their tolerance to this stress.

## 1. Introduction

Plant growth promoting microorganisms (PGPM) and their derivatives are key technology sources for sustainable agriculture, especially with the urgent need to slow climate change and its adverse effects (Naamala and Smith, 2020). For centuries, use of PGPM based inoculants to sustainably enhance plant growth and increase yield, under stressed and ideal conditions has been practiced in different parts of the world (Babalola and Glick, 2012; Bashan et al., 2014; García-García et al., 2020; Naamala et al., 2023). The ability of PGPM and or their derivatives to enhance plant growth is associated with their ability to exude in their growth environment, proteins and metabolites with plant growth promoting characteristics (Prithiviraj et al., 2003; Gray et al., 2006b; Schwinghamer et al., 2016; Piechulla et al., 2017).

Salinity stress is a major global constraint to crop production, affecting both plant yield quality and quantity. Although PGPM can mitigate the effects of salinity stress on plants, it can also affect the ability of PGPMs to enhance plant growth and may lead to microbial death in case of exposure to levels beyond those tolerated (Zahran, 1997; Soussi et al., 2001; Nadeem et al., 2015; Naamala et al., 2022, 2023). Some microbes have developed mechanisms for surviving at high salt concentrations. The ability of microbes to tolerate saline conditions is in part dependent on their ability to regulate salt concentration in their cytoplasm, in relation to that of their growth environment. Mechanisms employed to regulate salt concentration within the microbe include accumulation of osmolytes such as glutamate and proline in their cytoplasm through *de novo* synthesis or uptake from their growth environment (Zahran, 1997; Soussi et al., 2001; Oren, 2008; Bojanovic et al., 2017), upregulation of iron uptake mechanisms such as production of siderophores (Bojanovic et al., 2017), alteration of their cell membrane composition (Bojanovic et al., 2017; Hachicho et al., 2017), and maintenance of a high KCl concentration in their cytoplasm to match that of their growth medium (Oren, 2002; Oren, 2008). Effecting these mechanisms may necessitate the microbe to make changes to its genome, proteome, and metabolome profiles, most probably upregulating those components of each essential for enhancing salt tolerance mechanisms.

Protein expression occurs when genes are transcribed into messenger RNA (mRNA), which is then translated into proteins, which are major constituents of microbial cells (Karpievitch et al., 2010; Zhang et al., 2010). Protein expression and secretion are usually in response to either internal or external stimuli such as exposure to biotic and abiotic stress (Zhang et al., 2010; Armengaud et al., 2012; Schoof et al., 2022). The microbial proteome loosely translates to all proteins associated with a given microbe. The microbial exoproteome refers to proteins found in the immediate extracellular milieu of a microbe, arising from active cellular secretion, passive excretion and or cell lysis (Desvaux et al., 2010; Armengaud et al., 2012; Rubiano-Labrador et al., 2015; Schoof et al., 2022). For microbes cultured in laboratories, microbial exoproteome would refer to total proteins in spent media after removal of all microbial cells through centrifugation and filtration. Exoproteome composition reflects a microbe’s physiological state at a given time and can provide insight into a microbe’s interactions with its surroundings (Armengaud et al., 2012). Abiotic stresses such as salinity, acidity and alkalinity affect the quantity and quality of proteins synthesized and expressed by a microbe at a given time (Singleton et al., 1982; Soussi et al., 2001; Msimbira et al., 2022). Exploring the exoproteome of a microbe exposed to salt stress can provide insight into a set of proteins expressed in response to salt stress, which could then enhance our understanding of salt tolerance mechanisms in microbes (Rubiano-Labrador et al., 2015). In general, the ‘omics’ studies of biological systems have resulted in better understanding of microbes and their environment (Karpievitch et al., 2010). Advances in technology, such as invention of high through put tandem mass spectrometry and liquid chromatography have allowed for easy identification, analysis, classification, and function annotation of complex protein samples (Listgarten and Emili, 2005; Zhang et al., 2010; Armengaud, 2013; Kucharova and Wiker, 2014; Msimbira et al., 2022). It is interesting to note that while some PGPM may lose their ability to enhance plant growth following exposure to salt stress, others may gain or be more effective at enhancing plant growth, after exposure to some level of stress (Subramanian et al., 2021). Therefore, understanding how microbial exoproteome profiles change with changes in salt stress can improve utilization of CFS as plant growth biostimulants, and enhance our elucidation of mechanisms employed to enhance plant growth and or tolerate salt stress, given that some proteins such as enzymes play a vital role in stress tolerance and plant growth stimulation (Ahmad et al., 2010).

*Bacillus amyloliquefaciens* are rod shaped endospore forming gram positive bacteria from the genus *Bacillus* and family *Baciliaceae* (Woldemariam et al., 2020; Ngalimat et al., 2021). *B. amyloliquefaciens* is widely used in the food, pharmaceutical and agricultural sectors (Woldemariam et al., 2020). *B. amyloliquefaciens* and its derivatives have been reported to enhance plant growth under stressed and ideal conditions (Cappellari and Banchio, 2020; Cappellari et al., 2020; Duan et al., 2021; Kazerooni et al., 2021; Naamala et al., 2022). *Lactobacillus helveticus* is a gram positive facultative anaerobic lactic acid bacterium (LAB) that is widely used in the food processing industry. However, *L. helveticus* and its derivatives have also been reported to enhance plant growth (Naamala et al., 2023). This study was focused on understanding changes in exoproteome profiles of *B. amyloliquefaciens* EB2003A and *L. helveticus* EL2006H, at 0 mM NaCl and 200 mM NaCl. Results from previous studies have shown that CFSs of both strains, when exposed to 200 mM NaCl, enhanced germination and radicle length of soybean, and corn, as well as growth variables of potato (Naamala et al., 2022, 2023). We therefore point out some of the proteins identified in this study, that have been reported to enhance plant growth, as well as some of the mechanisms plants employ to support growth.

## 2. Materials and methods

### 2.1. Obtaining protein samples

*B. amyloliquefaciens* EB2003A and *L. helveticus* EL2006H, which were generously provided by EVL Inc., were cultured in 100 mL M13 and 100 mL De man, Rogosa and Sharpe (MRS) media, respectively, supplemented with 200 and 0 mM NaCl (control), at pH 7.0. They were incubated for 48 h (late exponential growth phase), at 120 rpm and a temperature of 30 and 37°C, for *B. amyloliquefaciens* EB2003A and *L. helveticus* EL2006H, respectively. Four replicates per treatment per species were cultured. Microbial cultures were then centrifuged, using a Sorvall Biofuge Pico (Mandel Scientific, Guelph, ON, Canada), for 10 min, at 10,000 rpm (15,180× g; SLA-1500) and 4°C, to pellet the microbial cells and separate them from the cell-free supernatant (CFS) (Gray et al., 2006a; Subramanian et al., 2021). The CFS was further vacuum filtered using 0.22 μm nylon filters to ensure that all bacterial cells were removed. Trichloroacetic acid (TCA; T9151, Sigma Aldrich) precipitation was used to extract total proteins from the obtained CFS replicates. The CFS was mixed with 100% (w/v) TCA, in 250 mL conical flasks to create a 25% working solution of TCA. The mixture was incubated at −20°C for 1 h, and transferred to an orbital shaker (MBI, Montreal Biotech Inc., Canada) with shaking speed of 90 rpm, in a cold room, at a temperature of - 4°C, for protein precipitation, overnight. This was followed by a 10 min centrifugation at 4°C and 10,000 rpm, to pellet the protein. The obtained protein pellet was then washed with ice-cold acetone, air-dried under a laminar flow hood, and dissolved in 2 M urea (U4883, Sigma Aldrich). The protein obtained from the four replicates of each treatment were pooled to form one sample per species. The experiment was repeated four times to get the appropriate biological replicates. Concentration of proteins obtained from the four experiments was determined, using the Lowry method (Lowry et al., 1951).

### 2.2. LC–MS/MS protein profiling

After determining the concentration of the obtained proteins, 10 μg protein per sample was dissolved in 20 μL of 2 M urea and sent to Montreal Clinical Research Institute (IRCM), for liquid chromatography mass spectrometry (LC–MS/MS) analysis. Total proteins were digested using trypsin enzyme and injected into an LC–MS/MS equipped with Linear Trap Quadrupole Velos Orbitrap (Thermo Fisher Waltham, MA, United States). The data set obtained from the mass spectra were searched against *Bacillus* spp. and *Lactobacillus spp.* databases, using Mascot software (Matrix Science, London, United Kingdom). Scaffold Software (version 5.1.2, Proteome Software Inc., Portland OR) was used to validate the obtained MS/MS based peptides and proteins, using an equal to or greater than 95% acceptance of protein probability, with a minimum of two peptides and 95% peptide probability (Keller et al., 2002).

### 2.3. Quantitative data analysis

Proteomic data for identified proteins obtained from the LC–MS/MS analysis was quantitatively analyzed, based on spectra count values, using Scaffold 5 (Scaffold Software for MS/MS Proteomics). Spectra count values were normalized and subjected to analysis of variance, at the 5% significance level, using a Benjamini-Hochberg multiple test correction, to detect significant differences between treatments. Significance was based on both fisher’s exact test (*p* ≤ 0.05) and fold change of more than or equal to 1.2. FASTA files generated from Scaffold 5 were analyzed using OmicsBox for functional annotation and interpretation of the protein sequences. Volcano plots were created using OriginPro software (OriginPro learning edition, version 2023 learning edition) while Venn diagrams were generated using Scaffold software for MS/MS proteomics. The LC–MS/MS proteomic data are available in the Mass Spectrometry Interactive Virtual Environment (*MassIVE*) at doi:10.25345/C5PG1HZ4M and PXD041778 for *Bacillus amyloliquefaciens* EB2003A, and doi:10.25345/C54B2XF6V and PXD041177 for *Lactobacillus helveticus*el2006h.

## 3. Results

### 3.1. Exoproteome analysis for *Bacillus amyloliquefaciens* EB2003A

**Figure 1:**
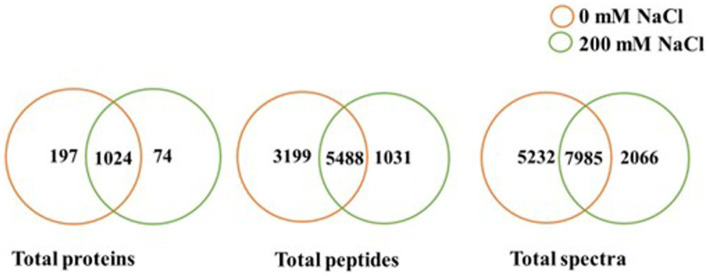
Comparison of total proteins, total peptides and total spectra identified at 0 and 200 mM NaCl, for *B. amyloliquefaciens* EB2003A (*p* ≤ 0.05).

Based on scaffold and OmicsBox analyzes of the LC–MS data, there were variations in identified proteins for CFS of *B. amyloliquefaciens* EB2003A cultured at 0 mM NaCl and 200 mM NaCl, as shown in Table 1 and Supplementary Table S1. In general, NaCl lowered the quantity of identified proteins, total unique spectra, and total unique peptides, as shown in Figure 1, visualized at 95% protein threshold, 2 minimum peptides and 0.00% decoy FDR. A total of 1,295 proteins, 9,718 total peptides and 15,283 total spectra were identified. Out of the observed proteins, 1,024 were shared between both salt levels while 197 were unique to 0 mM NaCl, and 74 proteins were unique to 200 mM NaCl.

**Table 1:** Comparing the distribution of *B. amyloliquefaciens* EB2003A proteins to the different functional groups according to GO enrichment analysis, at 0 mM NaCl (control) and 200 mM NaCl (treatment).

	# Sequences	
Functional group	0 mM NaCl	200 mM NaCl
Biological process		
Organic substance metabolic process	1,202	1,028 (↓14.5%)
Primary metabolic process	1,061	919 (↓13.4%)
Cellular metabolic process	1,100	912 (↓17.1%)
Nitrogen compound metabolic process	939	852 (↓9.3%)
Biosynthetic process	637	493 (↓22.6%)
Small molecule metabolic process	631	490 (↓22.3%)
Catabolic process	204	193 (↓5.4%)
Cellular components		
Intracellular anatomical structure	500	395 (↓21%)
Cytoplasm	468	363 (↓22.4%)
Membrane	162	**232 (↑30.2%)**
Cell periphery	99	**164 (↑39.6%)**
Intrinsic component of membrane	110	**159 (↑30.9%)**
Extracellular region	0	**19 (↑100%)**
Molecular function		
Ion binding	821	706 (↓14.0%)
Organic cyclic compound binding	701	620 (↓11.6%)
Heterocyclic compound binding	701	620 (↓11.6%)
Hydrolase activity	663	579 (↓12.7%)
Small molecule binding	580	463 (↓20.2%)
Oxidoreductase activity	438	331 (↓24.4%)
Transferase activity	481	354 (↓26.4%)
Carbohydrate derivative binding	350	296 (↓15.4%)
Catalytic activity, acting on a protein	177	**218 (↑18.8%)**
Catalytic activity, acting on a nucleic acid	160	**194 (↑17.5%)**
Ligase activity	54	0 (↓100%)
Enzyme code distribution		
Hydrolases	618	537 (↓13.1%)
Isomerases	114	90 (↓21.1%)
Ligases	182	148 (↓18.7%)
Lyases	134	102 (↓23.9%)
Transferases	463	344 (↓25.7%)
Translocases	54	**68 (↑20.6%)**
Oxidoreductases	418	310 (↓25.8%)

The bold values represent proteins upregulated at 200 mM NaCl.

**Figure 2:**
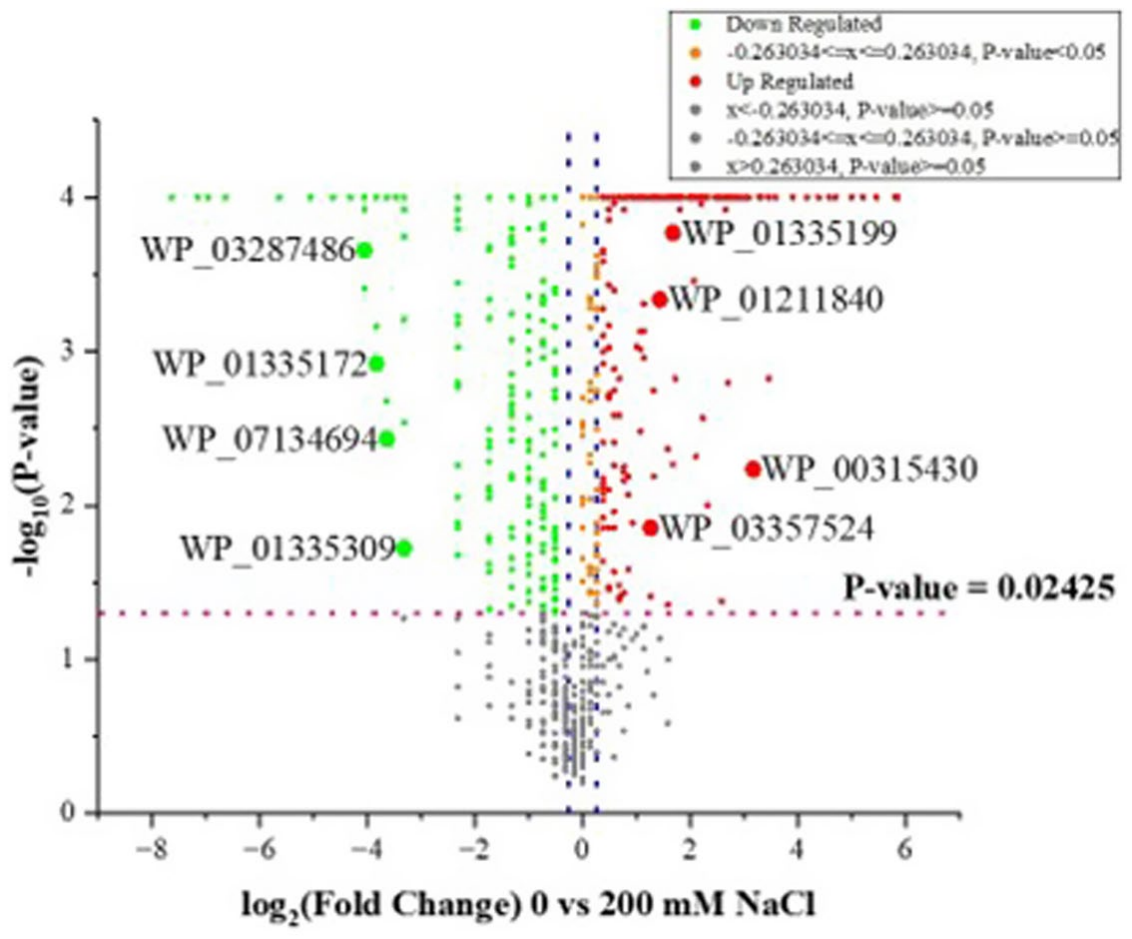
Volcano plots showing the distribution of identified proteins for *B. amyloliquefaciens* EB2003A as −log10 (Benjamini–Hochberg-adjusted *p*-values) plotted against log2 (fold change) for 0 vs. 200 mM NaCl. The two blue dotted vertical lines represent a ± 1.2-fold change, while the pink dotted horizontal line indicates the significance threshold (before logarithmic transformation) *p* ≤ 0.02425.

Further quantitative analysis of the LC–MS/MS data output using scaffold showed a significant decrease in the quantity of identified proteins at 200 mM NaCl in comparison to 0 mM NaCl, at *p* ≤ 0.05 (Fisher’s exact test). Several proteins were upregulated or down regulated at both salt levels as shown in Supplementary Table S1. Likewise, a number of proteins were unique to either 0 or 200 mM NaCl. Analysis with a ≥ 1.2 fold change also showed significant variations in proteins identified for 0 and 200 mM NaCl, as shown in Figure 2. Supplementary Table S3 shows OmicsBox data for *B. amyloliquefaciens* EB2003A.

### 3.2. Exoproteome analysis for *Lactobacillus helveticus* EL2006H

**Figure 3:**
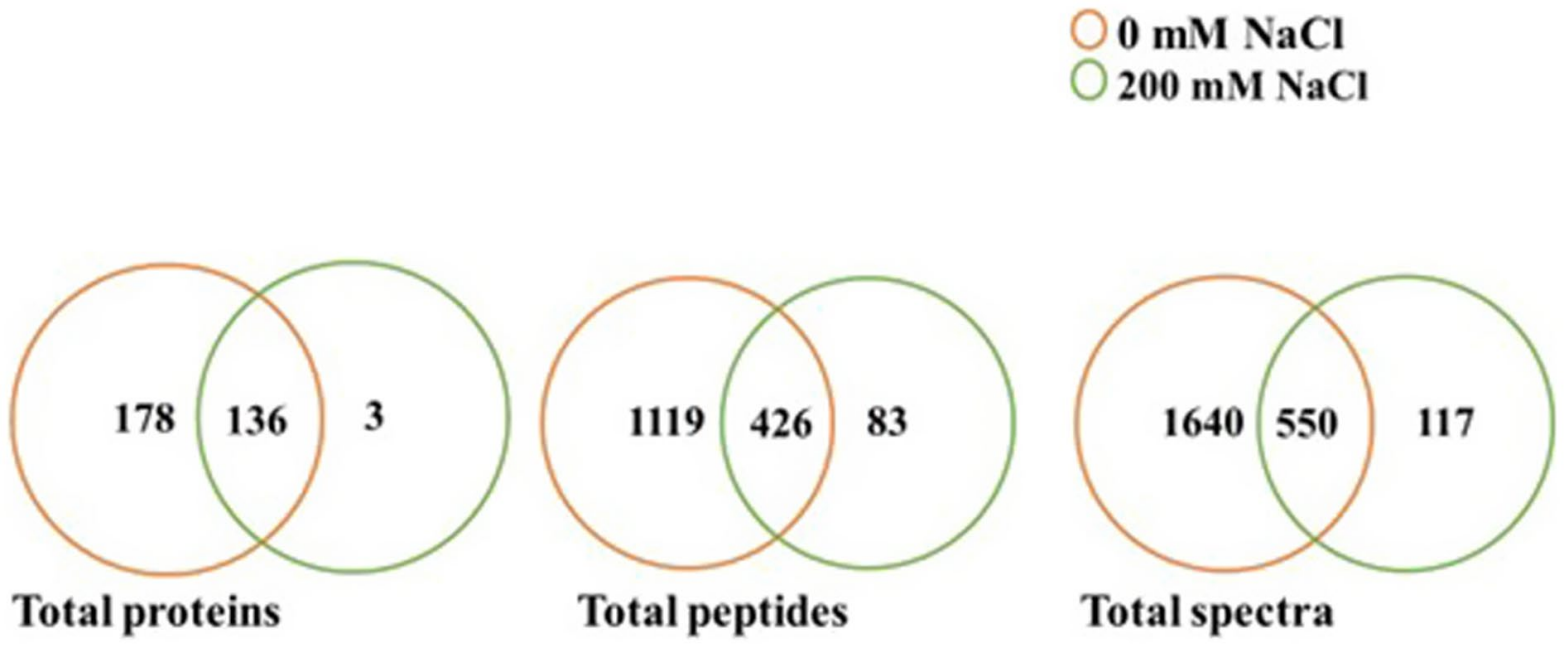
Comparison of total proteins, total peptides and total spectra identified at 0 and 200 mM NaCl for *L. helveticus* EL2006H (*p* ≤ 0.05).

Based on scaffold and OmicsBox analyzes of the LC–MS/MS data, there were variations in identified proteins for *L. helveticus* EL2006H cultured at 0 mM NaCl and 200 mM NaCl, as shown in Table 2 and Supplementary Table S2. Two hundred mM NaCl greatly affected identified proteins, with the majority downregulated, even to 100%. Figure 3 shows a comparison of the quantity of total proteins, total unique peptides, and total unique spectra for 0 and 200 mM NaCl, visualized at 95% protein threshold, 2 minimum peptides and 0.00% decoy FDR. A total of 317 proteins, 1,628 peptides and 2,307 spectra were observed. Out of the observed proteins, 136 were shared between both salt levels while 178 were unique to 0 mM NaCl, and 3 were unique to 200 mM NaCl, as shown in Figure 3.

**Table 2:** Comparing the distribution of *L. helveticus* EL2006H CFS proteins to the different functional groups according to GO enrichment analysis, at 0 mM NaCl (control) and 200 mM NaCl (treatment).

	# Sequences	
Functional group	0 mM NaCl	200 mM NaCl
Biological process		
Organic substance metabolic process	320	50 (↓84.4%)
Primary metabolic process	294	44 (↓85.0%)
Nitrogen compound metabolic process	282	46 (↓83.6%)
Cellular metabolic process	239	12 (↓94.9%)
Biosynthetic process	149	0 (↓100%)
Small molecule metabolic process	76	5 (↓93.4%)
Catabolic process	60	4 (↓93.3%)
Establishment of localization	62	51 (↓17.7%)
ATP metabolic process	8	0 (↓100%)
Transmembrane transport	0	**40 (↑100%)**
Cell adhesion	0	**7 (↑100%)**
Cellular components		
membrane	170	94 (↓44.7%)
intracellular anatomical structure	161	0 (↓100%)
intrinsic component of membrane	147	81 (↓44.8%)
cell periphery	110	75 (↓31.8%)
cytoplasm	92	0 (↓100%)
organelle	72	0 (↓100%)
extracellular region	17	**24 (↑29.2%)**
external encapsulating structure	29	24 (↓17.2%)
Transporter complex	0	**19 (↑100%)**
Membrane protein complex	0	**19 (↑100%)**
Molecular function		
Hydrolase activity	189	76 (↓59.7%)
Organic cyclic compound binding	200	7 (↓96.5%)
Heterocyclic compound binding	200	7 (↓96.5%)
Ion binding	138	14 (↓89.8%)
Catalytic activity, acting on a protein	89	39 (↓56.2%)
Small molecule binding	89	3 (↓96.6%)
Transferase activity	86	0 (↓100%)
Structural constituent of ribosome	67	0 (↓100%)
Carbohydrate derivative binding	53	0 (↓ 100%)
Isomerase activity	9	0 (↓100%)
ATP hydrolysis activity	18	0 (↓100%)
Peptidoglycan muralytic activity	0	**9 (↑ 100%)**
Protein binding	0	**16 (↑ 100%)**
Structural constituent of cell wall	0	**6 (↑ 100%)**
Enzyme code distribution		
Oxidoreductases	38	4 (↓89.5%)
Transferases	86	4 (↓95.3%)
Hydrolases	167	76 (↓54.5%)
Isomerases	33	3 (↓90.1%)
Translocases	9	6 (↓33.3%)
Lyases	18.5	0 (↓ 100%)
Ligases	21.75	0 (↓100%)

The bold values represent proteins upregulated at 200 mM NaCl.

**Figure 4:**
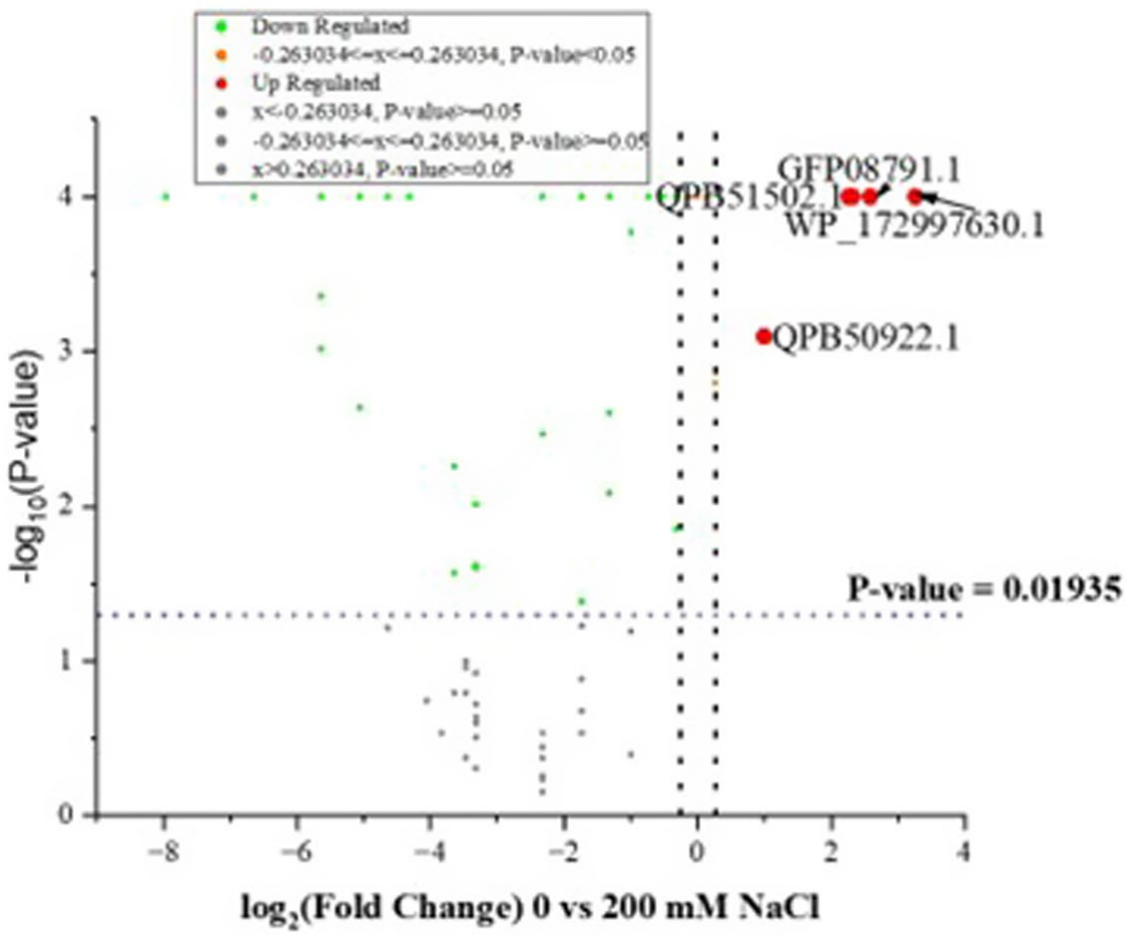
Volcano plots showing the distribution of identified proteins for *L. helveticus* EL2006H as −log10 (Benjamini–Hochberg-adjusted p-values) plotted against log2 (fold change) for 0 vs. 200 mM NaCl. The two dotted black vertical lines represent a ± 1.2-fold change, while the blue dotted horizontal line indicates the significance threshold (before logarithmic transformation) *p* ≤ 0.01935.

Further quantitative analysis of the LC–MS/MS output, using scaffold showed a significant decrease in identified proteins at 200 mM NaCl, in comparison to 0 mM NaCl, at *p* ≤ 0.05 (Fisher’s exact test). The majority of the proteins were significantly downregulated at 200 mM NaCl (Supplementary Table S2), with only seven upregulated proteins, namely, a cluster of hypothetical proteins GFB61_00500, a cluster of SLAP domain-containing proteins, a cluster of peptide ABC transporter substrate-binding proteins, surface proteins, fibronectin type III domain-containing proteins, a cluster of Stk1 family PASTA domain containing Ser/Thr kinases, and a cluster of metal ABC transporter substrate binding proteins. Based on the fold analysis (1.2-fold change and above), only three proteins namely, fibronectin type III domain-containing protein, cluster of hypothetical proteins and cluster of metal ABC transporter, were significantly upregulated as shown in Supplementary Table S2. Supplementary Table S4 shows OmicsBox data for *L. helveticus* 2006H. Figure 4 is a volcano plot illustrating the distribution of identified proteins for *L. helveticus* EL2006H.

### 3.3. Functional annotation of proteins of *Bacillus amyloliquefaciens* CFS observed at 0 and 200 mM NaCl

Using gene ontology (GO) enrichment analysis, identified proteins were grouped into four groups, based on their functions, namely, enzyme code distribution, cellular components, biological processes, and molecular functions, as shown in Table 1. There was a variation in the effect of 200 mM NaCl on the proteins performing various functions, majority being downregulated while some were upregulated, yet others were unique to 200 mM NaCl. Worthy noting is that all proteins involved in the various listed biological processes were downregulated while proteins related to the extracellular region cellular component were unique to 200 mM NaCl. Proteins involved in catalyzing proteins and nucleic acids were also upregulated by 18.8 and 17.5%, respectively. Under the enzyme code distribution, all enzymes were downregulated except for Translocases which were upregulated by 20.6%.

### 3.4. Functional annotation of proteins of *Lactobacillus helveticus* EL2006H CFS observed at 0 and 200 mM NaCl

Using gene ontology (GO) enrichment analysis, identified proteins were grouped in four sets, namely, biological processes, cellular components, molecular functions, and enzyme code distribution, as shown in Table 2. There was a variation in the effect of 200 mM NaCl on the proteins performing various functions; the majority were downregulated although a few were upregulated. Proteins in some functional groups were unique to 200 mM NaCl (Table 2). For example, under biological processes, transmembrane transport and cell adhesion were unique to 200 mM NaCl while under cellular components, transporter complex and membrane protein complex were unique to 200 mM NaCl. Under molecular function, peptidoglycan muralytic activity, protein binding and structural constituents of cell walls were unique to 200 mM NaCl. Notably, all the upregulated/unique proteins perform functions related to cell wall metabolism or substrate transportation.

## 4. Discussion

Uncontrollable changes in microbial environments, especially under field conditions, requires PGPM to adapt to the changes in order to survive (Gao et al., 2007; Galperin, 2010). Salinity stress is a leading global abiotic stress affecting crops and PGPM proliferation (Liu et al., 2023). When a microbe is exposed to stress, it may alter its proteome profile, upregulating proteins essential for enhancing tolerance to the stress while down regulating those that are likely not so essential (Galperin, 2010; Msimbira et al., 2022). As a result, proteome profiles of a microbe grown in different environmental conditions may vary significantly. The current study compared exoproteome profiles of *B. amyloliquefaciens* EB2003A and *L. helveticus* EL2006H exposed to 0 and 200 mM NaCl. Results of the study showed variations in total proteins identified for both strains at the two salt levels. Some of the identified proteins were unique to either 0 or the 200 mM NaCl. Among the proteins unique to 200 mM NaCl were cell wall metabolic enzymes, transcription/translation regulators, potential virulence factors, phage proteins, antibiotics resistance proteins, solute transporter proteins and, of course, hypothetical proteins. These findings are to some extent like those of Pumirat et al. (2009) and Rubiano-Labrador et al. (2015) who examined the exoproteome of *Burkholderia pseudomallei* and *Tistlia consotensis* exposed to salinity stress.

The cell-wall is the outermost layer of a bacterial cell, that acts as a stress barrier and maintains cell shape (Mueller and Levin, 2020). Therefore, maintaining the integrity of the cell wall is a mechanism for stress tolerance in bacteria. This may explain why, based on GO function analysis, the different functional groups identified in the current study, protein classes performing functions related to the cell wall and the extracellular region were upregulated at 200 mM NaCl, in both *B. amyloliquefaciens* EB2003A and *L. helveticus* EL2006H. Peptidoglycan is the major component of the cell wall, whose synthesis, polymerization, modification and turn over contribute to maintaining cell wall integrity (Popham and Young, 2003; Sauvage et al., 2008; Shin et al., 2020). In this study, proteins, such as LytR family transcriptional regulator, Amidases, peptidoglycan endopeptidases and penicillin binding proteins (PBPs), SLAP domain-containing protein and surface proteins were uniquely produced by either *B. amyloliquefaciens* EB2003A or *L. helveticus* EL2006H, exposed to 200 mM NaCl. They all play vital roles in peptidoglycan metabolism and maintenance of the cell wall. For example, LytR family transcriptional regulator proteins, also known as LytR-CpsA-Psr (LCP) family proteins, are in fact enzymes involved in the attachment of Glycopolymers, such as wall teichoic acids on the peptidoglycan (Kawai et al., 2011; Molloy, 2011; Gale et al., 2017; Siegel et al., 2019). Previously, this family of proteins was reported to play a transcription regulation role (Gao et al., 2007; Galperin, 2010), although in later studies, Kawai and co-authors disagreed (Kawai et al., 2011), suggesting that these regulatory roles could be bacterial genus, species, or strain specific. Bacterial amidases play a vital role in bacteria cell wall metabolism because, especially under stressful conditions, they are involved in the remodeling, turnover, recycling, and metabolism of peptidoglycan (Park, 1995; Weber et al., 2013; Senzani et al., 2017; Mueller and Levin, 2020). Endopeptidases play a major role in maintaining bacterial cell integrity and shape, through processes such as peptidoglycan turnover and modification (Shin et al., 2020). Penicillin binding protein PBP4, a peptidoglycan endopeptidase was reported to enhance tolerance of *B. subtilis* to salt stress by modifying the peptidoglycan (Palomino et al., 2009). *B. subtilis* was reported to recycle its peptidoglycan toward the end of its exponential growth, entering stationary phase, which could enable prolonged survival of the bacteria during stationary phase (Borisova et al., 2016). Surface proteins, also known as the glycoprotein layer or S layer proteins (Engelhardt, 2007; Hynönen and Palva, 2013), and surface layer associated proteins (SLAP), in the current study, were unique to *L. helveticus* treated with 200 mM NaCl. Expression of surface proteins has been linked to the ability of some *Lactobacilli* species to tolerate changes in the human gastrointestinal tract conditions, such as bile and low pH (Sengupta et al., 2013). It should be noted that, not all prokaryotes produce surface proteins and that their role varies from one group to another, leaving no universal function of surface proteins in species that do possess them (Engelhardt, 2007). Upregulation of surface proteins was also observed in *Lactobacillus acidophilus* IBB 801 exposed to different abiotic stresses such as NaCl, bile salt and high temperature (Grosu-Tudor et al., 2016). Deletion of *IgdA* SLAP resulted in a mutant that was more sensitive to salt stress and had a visibly disrupted cell surface when compared to strains with the protein (Klotz et al., 2020). However, the mechanisms through which surface proteins and SLAP enhance tolerance to salt stress is yet to be verified. Likewise, Stk1 family PASTA domain-containing Ser/Thr kinase also upregulated in *L. helveticus* exposed to 200 mM NaCl have been reported to play a role in bacteria cell wall metabolism and cell division (Janczarek et al., 2018). Its expression was reported to enhance tolerance of *Streptococcus suis* serotype 2 to oxidative stress (Zhu et al., 2014).

Exposure to stress may be a trigger for bacteria to reprogram their gene expression, consequently resulting in new gene products that could be essential for stress tolerance. In the current study, proteins such as MarR family transcriptional regulators and rRNA pseudouridine synthase were upregulated at 200 mM NaCl. The MarR family transcriptional regulators constitute a prominent family of transcription factors involved in the reprogramming of gene expression in response to stress conditions, such as oxidative stress (Pérez-Rueda et al., 2004; Grove, 2013; Deochand and Grove, 2017). MarR are involved in metabolism and antibiotic resistance of some bacteria (Will and Fang, 2020). The enzyme rRNA pseudouridine synthase catalyzes the synthesis of RNA pseudouridine from uracil, the most common modified nucleoside in rRNA that plays a role in gene expression (Ofengand, 2002; Zhao et al., 2018). Sulfate Transporter and Anti-Sigma factor antagonist (STAS) domain-containing proteins are produced in multiple species, including bacteria and mammals (Moy and Seshu, 2021). In bacteria, they are associated with stress tolerance among other factors, by regulating the large family of sigma factors (ρ) that bind to RNA polymerase to confer transcriptional target gene specificity (Moy and Seshu, 2021). For instance, sporulation in *B. subtilis*, which is a response to stress involves anti-anti-sigma factors, or anti-anti-ρ, which are STAS domain proteins (Sharma et al., 2011). Changes in gene products may also affect cellular biochemical composition and cellular processes, as observed in the current study.

Salt stress leads to osmotic, oxidative, and ionic stress in microbes, which, depending on their severity could cause damage to cellular components such as the cell membrane, nucleic acids, enzymes, and other proteins. A high concentration of salt ions such as Na^+^ and Cl^−^ in the microbe’s extracellular environment may cause loss of water from the microbial cell, leading to loss of cell turgor pressure as well as reduction in cellular processes such as metabolism (Krämer, 2010; Tsuzuki et al., 2011; Rubiano-Labrador et al., 2015). To counter act such effects, microbes develop mechanisms that enhance microbial tolerance to oxidative, osmotic, or ionic stress. In the current study, at 200 mM NaCl, proteins that enhance bacteria’s tolerance to oxidative stress were unique to 200 mM NaCl. For example, Thioredoxins play a major role in bacterial response to oxidative stress by quenching singlet oxygen, scavenging hydroxyl radicals and donating hydrogen to peroxidases (Chae et al., 1994; Das and Das, 2000; Zeller and Klug, 2006; Lu and Holmgren, 2014; Cheng et al., 2017). Members of the xenobiotic response element (XRE) family transcriptional regulators, among other functions, have been reported to enhance oxidative stress tolerance in different bacteria species such as *Streptococcus suis* and *Corynebacterium glutamicum* (Hu et al., 2019; Si et al., 2020; Zhang et al., 2022). Flavodoxin family proteins were reported to enhance tolerance of plant growth promoting rhizobacteria, such as *Pseudomonas fluorescens* Aur6 and *Ensiifer meliloti*, to oxidative stress (Coba de la Peña et al., 2013). Heme A synthase catalyzes the synthesis of heme A from heme O (Lewin and Hederstedt, 2016). Heme A is particularly a co-factor of terminal oxidase enzymes involved in oxygen reduction during aerobic respiration (Hederstedt, 2012; Choby and Skaar, 2016). High levels of intracellular heme have been reported to activate Hap1p which subsequently induces the transcription of genes involved in oxidative stress response (Martínez et al., 2016).

Since salt stress can result in damage to essential microbial constituents such as enzymes, and nucleic acids, DNA and RNA, it is important that damaged components are repaired or replaced with new ones. Upregulation of proteins that are directly or indirectly involved in synthesis and repair of cellular proteins and nucleic acids was observed in the current study. The enzyme 2′,3′-cyclic-nucleotide 2′-phosphodiesterase plays a major role in the metabolism of purines and pyrimidines, the building blocks of DNA and RNA, and provide energy and co-factors important in cell-division (Yin et al., 2018). This is because it contains cyclic phosphodiesterase and 3′-nucleotidase activity and catalyzes the hydrolysis of 2′,3′-cyclic nucleotides to yield nucleotides and phosphate. Therefore, the enzyme plays a role in DNA and RNA synthesis and repair through provision of building blocks. The enzyme m^1^A22-tRNA methyltransferase (TrmK) catalyzes N (1)-adenosine methylation to N1 of adenine 22 of bacterial tRNA (Roovers et al., 2008; Sweeney et al., 2022). Addition of a methyl group plays a role in maintaining stability of tRNA (Roovers et al., 2008). Stability of tRNA is essential in protein synthesis since they bridge the gap between mRNA and amino acids during translation. The enzyme thioredoxin plays a major role in protein repair and DNA synthesis by donating hydrogen that reduces ribonucleotide reductase and methionine sulfoxide reductase which catalyze the process (Zeller and Klug, 2006; Lu and Holmgren, 2014). The upregulation of such enzymes may also explain the high frequency of protein classes whose function annotation involves catalytic activity, acting on nucleic acid, and catalytic activity acting on proteins, that was observed in *B. amyloliquefaciens* EB2003A, at 200 mM NaCl.

When exposed to stress, its paramount that microbes maintain an even flow of substrates from their environment to the inside of the cell, and vice versa. This ensures availability of carbon sources for energy as well as metabolites required to serve purposes such as osmoregulation, enzyme co-factors, synthesis of proteins and nucleic acids. The microbe requires energy for channeling to survival mechanisms (Yan et al., 2015; Msimbira et al., 2022). Microbes respond to osmotic stress through intracellular accumulation of inorganic ions such as K^+^ and organic solutes such as proline (Soussi et al., 2001; Bojanovic et al., 2017). Although some of these osmo-protectants can be synthesized *de novo*, it is less energy efficient than sourcing them from outside of the cell (Zahran, 1997; Oren, 2008; Zhang et al., 2015; Bojanovic et al., 2017; Lycklama et al., 2018). Therefore, maintaining adequate substrate transport systems is essential for microbial tolerance to stress. In this study, we observed upregulation of a number of proteins associated with various substrate transport systems, such as the ATP binding cassette (ABC) transporter, the major facilitator superfamily (MFS) transporter and the phosphotransferase system (PTS) fructose transporter subunit IIC in *B. amyloliquefaciens* EB2003A and *L. helveticus* EL2006H. The ABC transporter facilitates transportation of a wide range of substrates such as sugars, amino acids, and metals from the microbe’s external environment (Du et al., 2011; Lycklama et al., 2018; Teichmann et al., 2018). Among the observed ABC transporter proteins were the amino acid ABC transporter substrate-binding protein, multispecies: ABC transporter substrate-binding protein, and multispecies: zinc ABC transporter substrate-binding protein. The MFS transporter is one of the oldest protein families, a group of secondary active transporters involved in selective transportation of substrates such as carbohydrates, amino acids, and lipids, to ions, peptides and nucleosides, across microbial membranes and plays a role in other microbial physiological processes, such as resistance to toxic compounds like antibiotics and salicylic acid, and enhanced salt tolerance (Yan, 2013; Lee et al., 2016; Pasqua et al., 2019). For instance, MFS efflux pumps VceCAB were reported to enhance the tolerance of *E. coli* to bile salts (Woolley et al., 2005). The PTS system is involved in uptake and phosphorylation of carbohydrates as well as signal transduction (Bernhard, 2012). The enzyme IIC component selectively transports sugar molecules across microbial membranes (Jahreis et al., 2008; Jason et al., 2014). This allows microbes such as bacteria to efficiently utilize carbohydrate sources of their choice, at a given time (Jahreis et al., 2008). The fructose family is a subfamily and the oldest of the glucose superfamily of PTS. The ability of an organism to utilize various carbon sources enables them to survive in varying environmental conditions. As a result, upregulation of sugar uptake systems has been linked to microbe response to stress, because microbes require nutrients and osmo-protectants for survival under stressful conditions (Pittman et al., 2014).

When exposed to stress, some members of the genus *Bacillus*, *B. amyloliquefaciens* EB2003A inclusive, form spores which are essential for survival under stressful conditions for long periods of time (Setlow, 2014; Ghosh et al., 2018). Once favorable conditions are restored, the spores germinate, giving rise to new microbial cells. The germination of spores is triggered by amino acids such as L-alanine, L- valine and L- asparagine (Setlow, 2014; Ghosh et al., 2018). In the current study, alanine containing proteins: cation symporter family protein and asparagine synthetase B were unique to the 200 mM NaCl exoproteome of *B. amyloliquefaciens* EB2003A. The enzyme asparagine synthase catalyzes the synthesis of asparagine from aspartate and glutamine (Lomelino et al., 2017; Zhu et al., 2019). The alanine cation symporter family protein is a transporter protein that transports alanine but no other amino acids (Ma et al., 2019).

In addition to the proteins with known functions, several hypothetical proteins were also unique to 200 mM NaCl treatment. Proteins are classified as hypothetical if a corresponding mRNA sequence is available in the data base, but there is no similar protein sequence, hence, insufficient information concerning their possible functions. However, it’s possible that such proteins play a role in enabling the microbe’s survival in growth conditions under which they are produced.

In previous studies, CFS of *B. amyloliquefaciens* EB2003A and *L. helveticus* EL2006H exposed to 200 mM NaCl enhanced germination and radicle length of corn and soybean and growth variables of potato grown under NaCl stress and normal conditions (Naamala et al., 2022, 2023). The ability of a microbe to enhance plant growth is related to its ability to exude into their growth environment, bioactive substances with ability to enhance plant growth. Among the proteins upregulated at 200 mM NaCl, in the current study, are those that have been reported to enhance plant growth under stressed and ideal conditions. Whether these proteins were in part responsible for the bioactivity observed in our previous study needs to be investigated further. However, application of exogenous heme has been reported to enhance plant tolerance to stress such salt stress (Woodson et al., 2011; Zhang et al., 2013; Wu et al., 2022). The heme precursor 5-aminolevulinic acid (ALA) was reported to enhance growth of plants exposed to salt stress (Hui et al., 2006; Daneshmand and Oloumi, 2015; Genişel and Erdal, 2016; Wu et al., 2022). Exogenous application of ALA, resulted in an increase in heme content, an indication that heme is involved in the role of ALA in alleviating salinity stress (Wu et al., 2022). Heme is involved in the transformation of superoxide anions in the antioxidant system, hence, potentially playing a pivotal role in mitigating the effects of oxidative stress on plants (Wu et al., 2022). Esterases have been reported to play a role in plant growth and development, involved in such crucial stages as seed germination, pollen development, lateral root, and overall root development (Takahashi et al., 2010; Clauss et al., 2011; Dolui and Vijayaraj, 2020; Zhang et al., 2020; Ursache et al., 2021; Shen et al., 2022). In fact, esterases are believed to have played a role in the evolutionary colonization of land by plants, through the conservation of water in a desiccating environment (Niklas et al., 2017; Philippe et al., 2020). MarR homologs were involved in symbiotic plant microbe interactions. For example, the MarR homolog ExpG *Sinorhizobium meliloti* activates transcription of three exp. operons that are involved in the production of galactoglucan, which it needs for plant root nodulation (Becker et al., 1997; Bartels et al., 2003). Proteins such as the MFS efflux pumps were reported to be involved in the interaction of plants and symbiotic microbes, such as rhizobia through enhancing nodulation and enhancing tolerance to flavonoids (Pasqua et al., 2019). Thioredoxin, another protein (enzyme) that was unique to 200 mM NaCl exoproteome of *B. amyloliquefaciens* EB2003A is also found in higher plants where it is classified as a disulfide regulatory protein, belonging to a complex of regulatory proteins consisting of types *f*, *m*, *x*, *y*, *h*, and *o* (Meng et al., 2010). Thioredoxin proteins play major roles in the regulation of carbon metabolism, embryogenesis, chloroplast development and mobilization of seed reserves, in plants (Jedelská et al., 2020). They also play a role in plant responses to biotic and abiotic stresses through protection from reactive oxygen species (Dos Santos and Rey, 2006; Meyer et al., 2012; Geigenberger et al., 2017). Thioredoxin *h* ortholog Trx *h*9, was reported play a role in the germination of wheat (Li et al., 2009). It is believed that Trx h regulates seed germination by reducing the disulfide proteins stored in the dry seed to the sulfhydryl state, following the addition of water (Maeda et al., 2003, 2005; Rhazi et al., 2003; Zahid et al., 2008). Meng et al. (2010) observed chlorotic leaves, short and smaller roots in *Arabidopsis thaliana Trx h*9 mutants. Furthermore, mutant plants were dwarf, with small and irregular mesophyll cells, as well as lower chloroplast numbers and less chlorophyll, in comparison to the wild type control, suggesting that Trx *h* plays a role in plant growth (Meng et al., 2010). High expression of thioredoxin h8 was observed in tobacco plants whose growth was enhanced when treated with *Bacillus aryabhattai* (Xu et al., 2022). Amidases are involved in the biosynthesis of indole acetic acid, a phytohormone that plays major roles in plant growth and development (Spaepen et al., 2007). Other PGPM such as *Pseudomonas putida* have also been reported to produce amidase (Chacko et al., 2009). Because they are involved in nitrogen metabolism, amidases increase nitrogen use efficiency in plants, which subsequently enhances plant growth under both stressed and non-stressed conditions (Unkefer et al., 2023).

There are several mechanisms through which the identified proteins can enhance plant growth. These include regulation of the anti-oxidant system, regulation of the photosynthetic system, ion balance, hydrolysis of compounds that affect plant quality, mobilization of nutrients during seed germination, biosynthesis of phytohormones involved in metabolic pathways such as nitrogen metabolism and maintenance of plant fertility (Spaepen et al., 2007; Takahashi et al., 2010; Clauss et al., 2011; Dolui and Vijayaraj, 2020; Zhang et al., 2020; Unkefer et al., 2023).

Based on these studies, it’s possible that some of the proteins upregulated at 200 mM NaCl stress were responsible for enhancing radicle length, germination of corn and soybean, and growth variables of potato, as observed in our previous studies.

## 5. Conclusion

Salinity stress affects survival, growth, and ability of PGPM to enhance plant growth. However, some PGPM have developed mechanisms of tolerating high levels of salt, altering their exoproteome profile being one. In the current study, *B. amyloliquefaciens* EB2003A and *L. helveticus* EL2006H, exhibited unique proteins when exposed to 200 mM NaCl, some of which have also been reported to enhance plant growth. Results of the study are in line with previous reports that when exposed to stress, microbes alter their exoproteome profile. To the best of our knowledge, this is the first study to report on the effect of NaCl on *B. amyloliquefaciens* EB2003A and *L. helveticus* EL2006H exoproteome profiles. Findings of this study will expand knowledge regarding mechanisms through which *Bacillus* spp. and *Lactobacillus spp.* tolerate salt stress at the protein level.

## Data availability statement

The datasets presented in this study can be found in online repositories. The names of the repository/repositories and accession number(s) can be found in the Mass Spectrometry Interactive Virtual Environment (MassIVE) online repository, at doi:10.25345/C5PG1HZ4M and PXD041778 for Bacillus amyloliquefaciens EB2003A, and doi:10.25345/C54B2XF6V and PXD041177 for Lactobacillus helveticusEL2006H.

## Author contributions

JN set up the experiments, was involved in data analysis and wrote the manuscript. SS advised on experimental set up, data analysis, and manuscript editing. LM helped with data analysis and manuscript review and editing. DS provided funding, the intellectual environment and did extensive manuscript editing. All authors have read and agreed to the final version of the manuscript.

## Funding

This work was funded through a grant from Consortium de recherche et innovations en bioprocédés industriels au Québec, number CRIBIQ 2017-034-C30, with support from synagri and EVL inc.

## Conflict of interest

The authors declare that the research was conducted in the absence of any commercial or financial relationships that could be construed as a potential conflict of interest.

## Publisher’s note

All claims expressed in this article are solely those of the authors and do not necessarily represent those of their affiliated organizations, or those of the publisher, the editors and the reviewers. Any product that may be evaluated in this article, or claim that may be made by its manufacturer, is not guaranteed or endorsed by the publisher.

## Supplementary material

SUPPLEMENTARY TABLE S1 Comparison of exoproteome for *B. amyloliquefaciens* EB2003A, at 0 and 200 mM NaCl.

SUPPLEMENTARY TABLE S2 Comparison of exoproteome for *L. helveticus* El2006H, at 0 and 200 mM NaCl.

SUPPLEMENTARY TABLE S3 *B. amyloliquefaciens* EB2003A OmicsBox data.

SUPPLEMENTARY TABLE S4 *L. helveticus* EL2006H OmicsBox data.
